# Variable Frequencies of Apolipoprotein E Genotypes and Its Effect on Serum Lipids in the Guangxi Zhuang and Han Children

**DOI:** 10.3390/ijms12095604

**Published:** 2011-08-31

**Authors:** Peng Hu, Yuan Han Qin, Feng Ying Lei, Juan Pei, Bo Hu, Ling Lu

**Affiliations:** 1 Department of Pediatrics, The First Affiliated Hospital of Anhui Medical University, No. 218 Jixi Road, Hefei 230022, China; E-Mails: hubo3218@sohu.com (B.H.); zbkllfdf@mail.hf.ah.cn (L.L.); 2 Department of Pediatrics, The First Affiliated Hospital of Guangxi Medical University, Nanning 530012, China; E-Mails: qinyuanhan603@yahoo.com.cn (Y.H.Q.); fenghuang0999@163.com (F.Y.L.); 3 Department of Nephrology, Peking University Third Hospital, Beijing 100083, China; E-Mail: pejoans@yahoo.com.cn

**Keywords:** apolipoprtein, ethnic group, genotype, total cholesterol, child

## Abstract

Guangxi Zhuang, the largest ethnic minority in China, is located in the southern part of the country, and well-known to the world as the longevity village. Studies of apolipoprotein E (APOE) polymorphism in adults suggest the lower frequencies of E4 allele and E4/E4 genotype may account, in part, for the favorable lipid profiles of Guangxi Zhuang. However, the effect of APOE polymorphism on serum lipids in the Guangxi Zhuang children is yet unknown to date. In the present study, genomic DNA was extracted from 278 Guangxi Zhuang and 200 Guangxi Han children. APOE genotypes were determined by PCR-restriction fragment length polymorphism (RFLP) analysis. The fasting serum lipoprotein a [Lp(a)], total cholesterol (TC), triglyceride (TG), high density lipoprotein cholesterol (HDL-C), low density lipoprotein cholesterol (LDL-C), apolipoprotein A1 (apoA1) and apoB were measured. Our results demonstrated that no significant differences in serum lipids were observed between the Guangxi Zhuang and Han children. The E4/E4 and E4/E3 genotypic frequencies were significantly lower in the Guangxi Zhuang children compared with the Guangxi Han children, whereas for E2/E2, E3/E2 and E4/E2 genotypic frequencies the opposite was presented. Though no significant differences in serum lipid concentrations were found for variant alleles both in the Guangxi Zhuang and Han children, the trend was observed in the association of higher levels of Lp(a), TC, TG and LDL-C with E4 allele in the Guangxi Zhuang children. In conclusion, a significant heterogeneity in APOE genetic variation indeed exists between the Guangxi Zhuang and Han ethnic group. The E4 allele may serve as a genetic marker for susceptibility to higher lipid profiles in the Guangxi Zhuang children. Lifestyle should be modified, according to APOE polymorphism even in the young children.

## 1. Introduction

The human apolipoprotein E (ApoE), a 34,200 kDa protein with 299 amino acids, is synthesized principally in the liver, but has also been found in other tissues such as the brain, spleen, kidneys, gonads, adrenals and macrophages [1,2]. The best recognized role of ApoE in lipid metabolism is as a ligand for receptor mediated clearance of chylomicron and very low density lipoprotein (VLDL) remnants [3]. Furthermore, it also participates in reverse cholesterol transport [4]. The APOE knockout mice exhibit the skyrocketing serum levels of total cholesterol (TC) and low density lipoprotein-cholesterol (LDL-C) similar to APOE deficient humans [5]. It has been estimated that about 14% variation in serum cholesterol is due to APOE polymorphism [6]. The polymorphic nature of APOE was established by Utermann and his associates [7]. The three major isoforms of ApoE, referred to as ApoE2, E3, and E4, are products of three alleles (E2, E3, and E4) at a single gene locus occurring on the chromosome 19q13.2. Three homozygous genotypes (E2/E2, E3/E3, and E4/E4) and three heterozygous genotypes (E3/E2, E4/E2, and E4/E3) arise from the expression of any two of the three alleles. The molecular basis of ApoE polymorphism is attributed to the amino acid substitutions in position 112 and 158 [8]. Among these genotypes, E3/E3 is the wild type and overwhelming in different populations with evident ethnic heterogeneity [9–11]. In general, individuals with the E2 allele tend to have lower levels, whereas individuals with E4 allele have higher levels of serum cholesterol in comparison to individuals who are homozygous for the E3 allele [12–14].

There are 56 ethnic groups in China. Han is the largest group, and Zhuang is the largest minority. One of China’s five autonomous regions, Guangxi Zhuang is located in the southern part of the country, and well-known to the world as the longevity village [15]. Its centenarian ratio stands at 24 per 100, 000 people, more than two times that of the United States and some other developed countries. Scores more nonagenarians and octogenarians also fill this village. Many of these elderly residents attribute the secret of their longevity to the *huo ma you,* a soup made from the oily seeds of the cannabis plant [16]. Besides this traditional diet, Guangxi Zhuang has some unique characteristics. It is geographically isolated, has a low immigration level, as well as strict intra-ethnic marriage. People dwelling in Guangxi Zhuang hold black color is beautiful, and they like to wear black garments and pants. It has preserved a unique linguistic, economic, and cultural identity. Compared to other Chinese regions, Guangxi Zhuang has a low genetic diversity [17].

The previous report of Ruixing *et al*. [18] documented that the levels of TC, triglyceride (TG), LDL-C, and apolipoprotein B (apoB) were significantly lower, whereas the levels of high density lipoprotein-cholesterol (HDL-C) and the ratio of apolipoprotein A1 (apoA1) to apoB were significantly higher in Guangxi Zhuang than in Han. More recently, another article in *Exp Biol Med* demonstrated that the frequencies of E4 allele and E4/E4 genotype were significantly lower in Guangxi Zhuang than in Han, and moreover, the E4 allele was significantly associated with higher TC and apoB levels both in the Guangxi Zhuang and Han adults. Therefore, the differences in the lipid profiles between Guangxi Zhuang and Han Chinese may partly attribute to the genetic variation of APOE [19]. However, subjects in both of the above studies were adults. The effect of APOE polymorphism on serum lipids in the Guangxi Zhuang and Han children is yet unknown to date. Next, the serum lipoprotein a [Lp(a)] level is generally assumed to be genetically determined for almost 90% of the value. It varies strongly from individual to individual and is elusive. In addition, to the best of our knowledge, little information exists about the association of apoE polymorphism with the Lp(a). In this context, we conducted the current study to probe the association of APOE polymorphism and lipid profiles in the Guangxi Zhuang and Han children.

## 2. Materials and Methods

### 2.1. Sample and Data Collection

A total of 278 Guangxi Zhuang children were randomly selected from two schools in Nanning city, the capital of Guangxi Zhuang Autonomous Region. Subjects ranged in age from 5 to 11 years, with an average age of 8.5 ± 2.4 years. There were 168 boys (60.4%) and 110 girls (39.6%). At the same time, a total of 200 Guangxi Han children residing in Nanning city were also recruited into our study. The mean age of the subjects was 8.3 ± 3.8 years (range 4 to 12). There were 132 boys (66.0%) and 68 girls (34.0%). The Ethics Committee of our Medical Faculty approved the study and samples were taken after obtaining informed consent from the parents of all subjects.

### 2.2. Laboratory Analysis

Blood samples for measuring serum lipid parameters were collected following an overnight fast. Serum was separated within 4 h and stored. Subsequent analysis of serum Lp(a) was performed using an assay based on “Sandwich” enzyme-linked immunosorbent assay (ELISA) that is insensitive to the presence of plasminogen (RAN-DOX, UK). The sensitivity and specificity of ELISA were found to be 98.2% and 95.7% respectively, and this test was not validated against the manufacture claims. Serum TC, TG, and HDL-C were measured by standard enzymatic method (RANDOX, UK). Serum LDL-C was calculated by the Friedewald formula [20]. Serum apoA1 and apoB were measured by immuno-turbidimetric methods (RANDOX, UK). All the analyses were performed in duplicate, and the examiners were blinded to the clinical and laboratory results.

### 2.3. APOE Genotyping

Blood for genotyping was drawn into EDTA-containing receptacles. Genomic DNA was prepared from peripheral blood leukocytes according to the standard procedure and stored at −4 °C [21]. PCR was performed according to the method reported by Seet *et al*. [22] using the primers: F 5′-TAAGCTTGGCACGGCTGTCCAAGGA-3′ and R 5′-ACAGAATTCGCCCCGGCCTGGTACAC-3′. The reaction’s mixture (total of 50 uL) was prepared with 10 pmol each primer, 100 Mm dNTPs, 1 U Taq polymerase (Promega, Madison WI, USA), buffer containing 1.5 mM MgCl_2_, and 500 ng DNA of the sample. The amplification process consisted of an initial denaturation at 95 °C for 5 min, followed by 30 cycles of denaturation at 95 °C for 1 min, coiling at 61 °C for 1 min, extension at 70 °C for 1 min, and a final extension at 72 °C for 10 min. Negative controls (no DNA added) were included in every PCR run to check for contamination. The efficacy of the PCR was visualized through electrophoresis on 2% agarose gel with ethidium bromide, when a band of 240 bp was observed under ultraviolet light. Genotyping was performed by digesting the PCR product (20 uL) overnight with 5 U of the restriction enzyme Hha I (Takara, Dalian, China). The fragment sizes from polymorphic Hha I sites after cleavage were as follows: E2/E2: 91 and 83 bp; E3/E3: 91, 48, and 35 bp; E4/E4: 72, 48, and 35 bp; E3/E2: 91, 83, and 48 bp; E4/E2: 91, 83, 72, and 48 bp; E4/E3: 91, 72, and 48 bp.

### 2.4. Statistical Analysis

The results are reported as means ± standard deviations or percentages. The allelic frequencies and genotype distribution were calculated by the gene-counting method [23]. The *X*^2^-test or Fisher’s exact test was used to evaluate the allelic and genotypic frequencies, and to also estimate the Hardy-Weinberg equilibrium. One-way analysis of variance (ANOVA) and Student-Newman-Keuls post test were performed to determine the differences of lipid parameters among genotypes and alleles. A value of *p* < 0.05 was considered as significant. Statistical analysis was performed using the statistical package for social studies SPSS version 11.5.

## 3. Results

The general characteristics and serum lipids of two groups in this study are shown in Table 1. Male/female ratio, age, body mass index (BMI), systolic blood pressure (SBP), diastolic blood pressure (DBP), and pulse pressure (PP) were almost identical in the two groups (*p* > 0.05). Similarly, no significant differences in serum lipids were observed between the Guangxi Zhuang and Han children (*p* > 0.05).

The frequencies of APOE genotypes and alleles in Guangxi Zhang and Han children are shown in Table 2. The genotype distribution did not deviate from Hardy-Weinberg equilibrium for the Guangxi Zhuang and Han children (*p* > 0.05). The E3/E3 was observed to be the most common genotype in the Guangxi Zhuang children (69.4%) as well as in the Guangxi Han Children (73.0%). E2/E2 genotype was not present in the Guangxi Han children. The E4/E4 and E4/E3 genotypic frequencies were significantly lower in the Guangxi Zhuang children compared with the Guangxi Han children, whereas for E2/E2, E3/E2 and E4/E2 genotypic frequencies the opposite was present (*p* < 0.05). The E3 allele was the most prevalent form both in the Guangxi Zhuang (82.0%) and Han children (83.0%), respectively. The E4 allelic frequency was found to be marginally lower, on the contrary, E2 allelic frequency was slightly higher in the Guangxi Zhuang children in comparison with the Guangxi Han children, but these differences did not reach statistical significance (*p* > 0.05).

**Figure 1:**
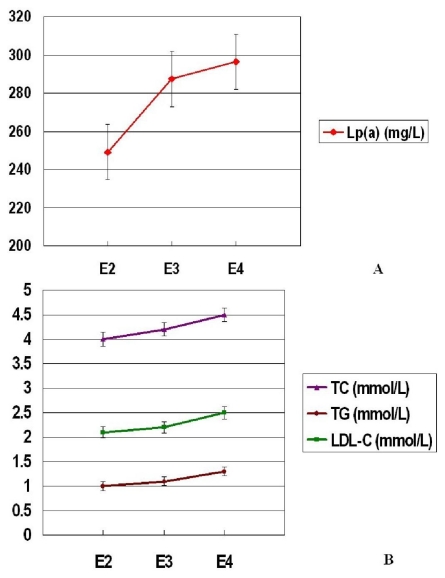
The influence of APOE alleles on serum lipid concentrations in the Guangxi Zhuang children. Though no significant differences in serum lipid concentrations were found for variant alleles (*p* > 0.05), the trend was observed in the association of higher levels of Lp(a), TC, TG and LDL-C with E4 allele in the Guangxi Zhuang children.

The influences of APOE genotypes and alleles on serum lipid concentrations in two groups are shown in Table 3. No significant differences in serum lipid concentrations were observed for variant genotypes both in the Guangxi Zhuang and Han children (*p* > 0.05). To evaluate the association of serum lipids with the APOE alleles, we divided subjects in three subgroups: E2 carriers (E2/E2 and E3/E2), E3 carriers (E3/E3), and E4 carriers (E4/E4 and E4/E3). Individuals with E4/E2 were not included in analysis because of the uncertainy about pooling with other genotypes. Though no significant differences in serum lipid concentrations were found for variant alleles both in the Guangxi Zhuang and Han children (*p* > 0.05), the trend was observed in the association of higher levels of Lp(a), TC, TG and LDL-C with E4 allele in the Guangxi Zhuang children (Figure 1). The influences of sex, age, and BMI on lipid metabolism have also been taken into account in this study. First, the present study was performed in prepubetal children, in order to avoid the hormonal and gender interventions to lipid metabolism. Second, all subjects recruited into our study were healthy children, and individuals with overweight or obesity (BMI > 24 kg/m^2^) were excluded. Third, the general characteristics, including male/female ratio, age, BMI, SBP, DBP, and PP, were almost statistically identical in the two groups. Given this background, despite of unadjusted values, statistical analyses in the current study can reflect the true situation without any discount.

**Figure 2:**
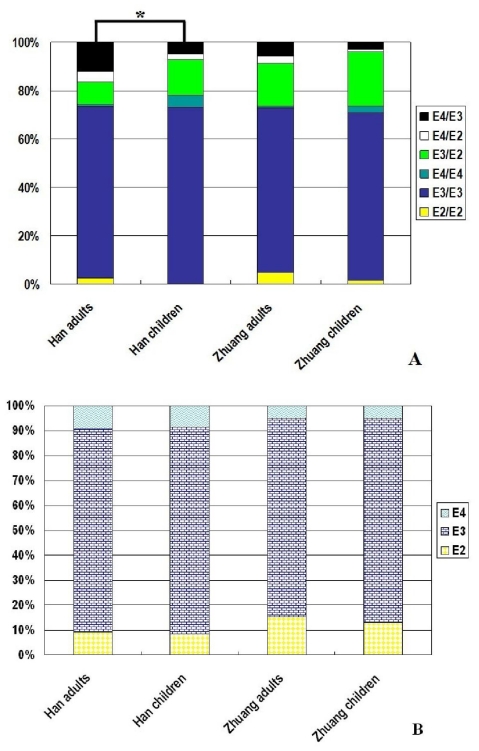
The comparative analysis of APOE polymorphism between the Guangxi adults and children. The genotypic distribution in the Guangxi Han adults significantly differed from the Guangxi Han children (* *p* < 0.05).

The comparative analysis of APOE polymorphism between the Guangxi adults and children are shown in Figure 2. According to the data of Yin *et al*. [19], the frequencies of E2/E2, E3/E2, E4/E2, E3/E3, E4/E3, and E4/E4 genotypes were 4.7%, 17.9%, 3.2%, 68.2%, 5.5%, and 0.6% in the Guangxi Zhuang adults, and 2.5%, 9.2%, 4.2%, 70.7%, 12.2%, and 1.2% in the Guangxi Han adults, respectively. The frequencies of E2, E3, and E4 alleles were 15.2%, 79.8%, and 4.9% in the Guangxi Zhuang adults, and 9.2%, 81.4%, and 9.3% in the Guangxi Han adults, respectively. The genotypic distribution in the Guangxi Han adults significantly differed from the Guangxi Han children (*p* < 0.05).

## 4. Discussion

The prevalence of cardiovascular disease (CVD) in adulthood is a thorny public health issue throughout the world. The CVD process is considered to start early in life and has been causally linked to elevated levels of serum lipids since childhood [24]. The study of individual differences in the early dyslipidemia and progression of potential initiating risk factors is very important. The lipid profiles of children are scarcely influenced by environmental factors, such as smoking and insobriety, thereby may be more dependent on the genetic basis than that of adults [25]. APOE has been identified as an important candidate gene for lipid abnormalities [26]. The association of APOE polymorphism with serum lipids is highly explored in adults, but not in children, particularly the Guangxi Zhuang and Han children.

The present study was performed in prepubetal children, in order to avoid the hormonal and gender interventions to lipid metabolism. In addition, other confounding factors, such as BMI, SBP, DBP and PP, were almost identical between the Guangxi Zhuang and Han children. As a result, our data demonstrated that no significant differences in serum lipids were observed between the Guangxi Zhuang and Han children. However, the previous report of Ruixin *et al*. [18] documented that the levels of TC, TG, LDL-C, and apoB were significantly lower, whereas the levels of HDL-C and the ratio of apoA1 to apoB were significantly higher in the Guangxi Zhuang adults, compared to the Guangxi Han adults. We hypothesized that physiological shift and multiple environmental factors may contribute, in part, to these disagreements.

In subsequent analysis, all six APOE genotypes were detected in our subjects, and their frequencies were E2/E2: 1.4%, E3/E3: 69.4%, E4/E4: 2.9%, E3/E2: 22.3%, E4/E2: 1.1%, and E4/E3: 2.9% in the Guangxi Zhuang children; E3/E3: 73.0%, E4/E4: 5.0%, E3/E2: 15.0%, E4/E2: 2.0%, and E4/E3: 5.0% in the Guangxi Han children, respectively. The frequencies of APOE alleles were E2: 13.1%, E3: 82.0%, and E4: 4.9% in the Guangxi Zhuang children; E2: 8.5%, E3: 83.0%, and E4: 8.5% in the Guangxi Han children, respectively, estimated by the gene-counting method. The E4/E4 and E4/E3 genotypic frequencies were significantly lower in the Guangxi Zhuang children compared with the Guangxi Han children, whereas for E2/E2, E3/E2 and E4/E2 genotypic frequencies the opposite was presented. Furthermore, the E4 allelic frequency was found to be marginally lower, on the contrary, E2 allelic frequency was slightly higher in the Guangxi Zhuang children in comparison with the Guangxi Han children, but these differences did not reach statistical significance. These above findings in our study were consistent with the previous research on the Guangxi Zhuang and Han adults [19]. Therefore, a significant heterogeneity in APOE genetic polymorphism indeed exists between the Guangxi Zhuang and Han ethnic group.

Despite this consensus, questions remain as to whether the frequencies of APOE genotypes and alleles are similar between adults and children even within the same ethnic group. In the present study, we further conducted the comparative analysis of APOE polymorphism between the Guangxi adults and children, and found that the genotypic distribution in the Guangxi Han adults significantly differed from the Guangxi Han children. More specifically, the E4/E4 genotypic frequency was significant lower in the Guangxi Han adults than did in the Guangxi Han children (1.2% *versus* 5.0%). It is not a unique instance, many cohort studies have also demonstrated that genetic polymorphism of APOE is associated with human longevity. The E4/E4 genotypic frequency in olds is the lowest, which attributes to the susceptibility to CVD in E4/E4 carriers [27–29].

An important part of the current study was to elucidate the influences of APOE genotypes and alleles on serum lipid concentrations in the Guangxi Zhuang and Han children. Though no significant differences in serum lipid concentrations were observed for variant genotypes and alleles both in the Guangxi Zhuang and Han children, the trend was observed in the association of higher levels of Lp(a), TC, TG and LDL-C with E4 allele in the Guangxi Zhuang children. However, in the report of Yin *et al*. [19], the E4 allele was significantly associated with higher TC and apoB levels both in the Guangxi Zhuang and Han adults. We postulated that the relative small sample size and environmental factors may act as the main triggers leading to the different consequences of APOE genetic polymorphism on the childhood lipid profiles as compared to that in adults. More persuasively, the results of a 21-year longitudinal study on changes in serum lipids in 1233 Finns followed from childhood to adulthood consistently observed the E4 allele to be associated with higher TC and LDL-C levels in childhood. The LDL-C-elevating effect of the E4 allele was an association that was tracked through to adulthood, having a greater effect with increasing age [30]. Therefore, even in the young children, the E4 allele may be potential in predicting their lifelong exposure to an adverse lipid profiles, and lifestyle modifications (mainly by practicing regular physical activity and eating a healthy diet) should be highly encouraged in such children [31,32].

## 5. Conclusions

A significant heterogeneity in APOE genetic polymorphism indeed exists between the Guangxi Zhuang and Han ethnic group. The E4 allele may serve as a genetic marker for susceptibility to higher lipid profiles in the Guangxi Zhuang children. Lifestyle should be modified, according to the genetic polymorphism of APOE even in the young children.

## Figures and Tables

**Table 1:** The general characteristics and serum lipids of the Guangxi Zhuang and Han children.

Group	Guangxi Zhuang children (*n* = 278)	Guangxi Han children (*n* = 200)	*p*-value
Male/female	168/110	132/68	NS
Age (years)	8.5 ± 2.4	8.3 ± 3.8	NS
BMI (kg/m^2^)	15.3 ± 4.2	15.8 ± 3.6	NS
SBP (mmHg)	98.6 ± 9.5	100.7 ± 10.2	NS
DBP (mmHg)	65.8 ± 9.1	66.5 ± 8.4	NS
PP (mmHg)	34.7 ± 7.9	37.9 ± 8.7	NS
Lp(a) (mg/L)	246.0 ± 42.7	277.5 ± 37.7	NS
TC (mmol/L)	4.1 ± 0.8	4.5 ± 0.4	NS
TG (mmol/L)	1.1 ± 0.4	0.9 ± 0.3	NS
HDL-C (mmol/L)	1.5 ± 0.3	1.4 ± 0.1	NS
LDL-C (mmol/L)	2.2 ± 0.6	2.1 ± 0.4	NS
apoA1 (g/L)	1.5 ± 0.3	1.2 ± 0.1	NS
apoB (g/L)	0.8 ± 0.3	0.9 ± 0.3	NS
apoA1/B	1.7 ± 0.5	1.4 ± 0.4	NS

NS, Not significant.

**Table 2:** The frequencies of APOE genotypes and alleles in the Guangxi Zhang and Han children.

Genotype or allele	Guangxi Zhuang children *n* = 278 (%)	Guangxi Han children *n* = 200 (%)	*X^2^*	*p*-value
E2/E2	4 (1.4)	0 (0)		
E3/E3	193 (69.4)	146 (73.0)		
E4/E4	8 (2.9)	10 (5.0)		
E3/E2	62 (22.3)	30 (15.0)		
E4/E2	3 (1.1)	4 (2.0)		
E4/E3	8 (2.9)	10 (5.0)	11.25	0.04
E2 allele	36.5 (13.1)	17 (8.5)		
E3 allele	228 (82.0)	166 (83.0)		
E4 allele	13.5 (4.9)	17 (8.5)	4.59	0.10

**Table 3:** The influences of APOE genotypes and alleles on serum lipid concentrations in the Guangxi Zhuang and Han children.

	E2/E2	E3/E3	E4/E4	E3/E2	E4/E2	E4/E3	E2	E3	E4
**Guangxi Zhuang children**									
*N* = 278	4	193	8	62	3	8	36.5	228	13.5
Lp(a) (mg/L)	233.7 ± 39.1	287.5 ± 40.6	298.3 ± 56.5	264.3 ± 35.7	300.6 ± 48.9294.2 ± 36.8	249.1 ± 37.5	287.5 ± 40.6	296.3 ± 45.9	
TC (mmol/L)	3.9 ± 0.9	4.2 ± 0.5	4.5 ± 0.7	4.0 ± 0.5	4.5 ± 0.8	4.3 ± 0.6	4.0 ± 0.7	4.2 ± 0.5	4.5 ± 0.7
TG (mmol/L)	0.9 ± 0.5	1.1 ± 0.3	1.3 ± 0.4	1.1 ± 0.4	1.2 ± 0.5	1.2 ± 0.4	1.0 ± 0.4	1.1 ± 0.3	1.3 ± 0.3
HDL-C (mmol/L)	1.3 ± 0.6	1.6 ± 0.3	1.6 ± 0.5	1.5 ± 0.4	1.5 ± 0.6	1.5 ± 0.4	1.4 ± 0.4	1.6 ± 0.3	1.5 ± 0.5
LDL-C (mmol/L)	2.0 ± 0.7	2.2 ± 0.4	2.5 ± 0.9	2.1 ± 0.6	2.3 ± 0.8	2.5 ± 0.7	2.1 ± 0.6	2.2 ± 0.4	2.5 ± 0.7
apoA1 (g/L)	1.4 ± 0.4	1.4 ± 0.3	1.6 ± 0.5	1.5 ± 0.3	1.4 ± 0.5	1.5 ± 0.4	1.5 ± 0.4	1.4 ± 0.3	1.5 ± 0.4
apoB (g/L)	0.7 ± 0.3	0.8 ± 0.2	0.8 ± 0.3	0.8 ± 0.3	0.8 ± 0.4	0.9 ± 0.4	0.8 ± 0.3	0.8 ± 0.2	0.8 ± 0.3
apoA1/B	1.6 ± 0.5	1.7 ± 0.5	1.7 ± 0.6	1.7 ± 0.5	1.6 ± 0.6	1.7 ± 0.51.7 ± 0.5	1.7 ± 0.5	1.7 ± 0.6	
**Guangxi Han children**									
*n* = 200	0	146	10	30	4	10	17	166	17
Lp(a) (mg/L)		236.5 ± 34.1	306 ± 43.2	296.2 ± 33.4	311.3 ± 52.1	305 ± 32.4	296.2 ± 33.4	236.5 ± 34.1	305.3 ± 41.5
TC (mmol/L)		4.1 ± 0.8	4.9 ± 1.3	4.3 ± 0.6	4.9 ± 2.1	4.5 ± 1.6	4.3 ± 0.6	4.1 ± 0.8	4.7 ± 1.1
TG (mmol/L)		0.8 ± 0.2	1.0 ± 0.4	0.9 ± 0.2	0.9 ± 0.5	0.8 ± 0.3	0.9 ± 0.2	0.8 ± 0.2	0.9 ± 0.3
HDL-C (mmol/L)		1.3 ± 0.7	1.7 ± 0.8	1.5 ± 0.6	1.3 ± 0.7	1.5 ± 0.8	1.3 ± 0.7	1.3 ± 0.7	1.6 ± 0.8
LDL-C (mmol/L)		1.9 ± 0.3	2.4 ± 0.7	2.2 ± 0.5	2.2 ± 1.0	2.3 ± 0.6	2.2 ± 1.0	1.9 ± 0.3	2.3 ± 0.6
apoA1 (g/L)		1.1 ± 0.1	1.3 ± 0.3	1.2 ± 0.3	1.4 ± 0.5	1.3 ± 0.4	1.2 ± 0.3	1.1 ± 0.1	1.3 ± 0.3
apoB (g/L)		0.8 ± 0.2	1.2 ± 0.4	0.8 ± 0.2	1.0 ± 0.4	1.1 ± 0.4	0.8 ± 0.2	0.8 ± 0.2	1.1 ± 0.4
apoA1/B		1.4 ± 0.2	1.1 ± 0.4	1.4 ± 0.3	1.4 ± 0.3	1.2 ± 0.4	1.4 ± 0.31.4 ± 0.2	1.2 ± 0.4	

No significant differences in serum lipid concentrations were observed for variant genotypes and alleles both in the Guangxi Zhuang and Han children (*p*> 0.05).
